# How to Formulate for Structure and Texture via Medium of Additive Manufacturing-A Review

**DOI:** 10.3390/foods9040497

**Published:** 2020-04-15

**Authors:** Azarmidokht Gholamipour-Shirazi, Michael-Alex Kamlow, Ian T. Norton, Tom Mills

**Affiliations:** School of Chemical Engineering, University of Birmingham, Edgbaston, Birmingham B15 2TT, UK; MXK871@student.bham.ac.uk (M.-A.K.); I.T.NORTON@bham.ac.uk (I.T.N.); t.b.mills@bham.ac.uk (T.M.)

**Keywords:** additive manufacturing, 3D food printing, extrusion, Inkjet, customized food, hydrocolloids

## Abstract

Additive manufacturing, which is also known as 3D printing, is an emerging and growing technology. It is providing significant innovations and improvements in many areas such as engineering, production, medicine, and more. 3D food printing is an area of great promise to provide an indulgence or entertaining experience, personalized food product, or specific nutritional needs. This paper reviews the additive manufacturing methods and materials in detail as well as their advantages and disadvantages. After a full discussion of 3D food printing, the reports on edible printed materials are briefly presented and discussed. In the end, the current and future outlook of additive manufacturing in the food industry is shown.

## 1. Introduction

The term “additive manufacturing (AM)”, which is often termed 3D printing and rapid prototyping, covers several quite different manufacturing technologies that enable the creation of objects to be fabricated on demand [1,2]. The basic concept of AM is a controlled process whereby a product is built up from a digital design (usually a Computer-Aided Design (CAD) file) [3,4]. While printing the object, the 3D model is sliced into layers, by the software, and then printed one at a time (layer by layer) [5]. AM has emerged as one of the most disruptive manufacturing technologies and, consequently, has significant implications for companies and industries [6]. The adoption of these technologies comprises four successive stages: rapid prototyping, rapid tooling, digital manufacturing, and home fabrication [5]. Each stage corresponds to a different degree of involvement of 3D printing in the manufacturing process. By using a business model, Rayna and Striukova [5] have concluded that rapid prototyping and rapid tooling had a limited impact, but direct manufacturing and home fabrication can be highly disruptive [5].

The advantages of AM, in general, are low waste, increased precision, time-saving, and high efficiency [7]. Table S1 collects a series of benefits and shortcomings alongside potential economic effects. AM is a platform that enables the manufacture of complex structures from digital design data immediately, without special tools and equipment, by providing new opportunities for freedom of design [8]. Rapid prototyping is one of the main benefits of 3D printing [9]. AM offers the capacity to build objects with any shape and dimension as well as the ability to control chemical, physical, mechanical properties, and an internal structure (microstructure) by modifying their composition [10,11].

AM is a mass-customisation and personalisation-enabling technology [6]. Due to limits in production speed among other technological bottlenecks, 3D printing is ideal for mass customisation but is not yet capable of mass production [6,12]. AM enables private and commercial users to design and create their products simply and quickly, especially for low volume, customised, high-value products [8,13]. Most equipment used in AM can be used to produce a variety of different parts at nearly any location by potentially allowing decentralisation and localisation of production for companies [6].

Therefore, AM offers opportunities for advancement in remote areas with low economic profiles by bridging the gap between these areas and the next market, and by supplying these areas with the necessary objects to improve quality of life [13]. AM significantly lowers labour demands as well as financial and energy resource inputs into manufacturing processes, which leads to a decrease in production costs and CO_2_ emissions [13]. The layer-by-layer nature of production, specifically the ability to lower product infill, greatly lowers resource demands and process-related waste [13]. Conventionally, one-of-a-kind manufacturing is associated with surplus material being wasted [8]. However, with AM technologies, highly customised products can be manufactured more sustainably [1]. By using 3D printing, it could be possible to develop a local material recycling and manufacturing loop, which translates into reductions in landfill and emissions, and leads to an increase in local employment through recycling centres as well as value creation [14].

In its early days, AM was used for rapid prototyping and then tooling and these application areas continue to be used [1]. There is an abundance of evidence that suggests AM will be promising in the following areas [15,16]: (1) customised healthcare products for improving health and quality of life. There are several reports on amenable 3D printing technologies for pharmaceutical manufacturing [17,18], which are applicable to different drug development phases (early-phase screening, testing, manufacturing, and dispensing) [19]. Human medicine, as well as veterinary medicine, can benefit from technological advances in 3D printing [20,21,22,23,24]. 3D printing is important to dentistry and can be used to print personalised braces for patients [25,26]. Surgeons have also reported the application of 3D printing in their respective subspecialties [27,28,29]. (2) Other evidence that shows AM is promising includes reduced environmental impact by increasing manufacturing sustainability. In the construction industry, many experiments have been conducted to explore the full potential of 3D printing as a core method to build more sustainable and environmentally-friendly buildings, or to produce construction components [30]. Considerable research is ongoing to use wood and forest products as 3D printing feedstock to impart texture to the surface of the printed products and meet the demand for sustainability from consumers [12]. (3) The third piece of evidence is supply chain simplification to increase efficiency and responsiveness. The various AM technologies that enable the development of fashion products like apparel and jewelry are closely associated with low-volume manufacturing and mass customization [31].

Additive manufacturing has been labelled a “disruptive technology” because it will fundamentally affect many processes in production, supply chain design, logistics, product life-cycle planning, and consumer behavior [8]. Due to the performance improvements of AM technologies, they are seeing increased usage in direct manufacturing rather than being classified as a ‘technique for prototyping’ [10]. For example, 3D printers are being utilised in analytical laboratories in a wide array of applications [32]. Moreover, the societal impact study from a technical perspective shows further research is necessary in life-cycle energy consumption evaluation and potential occupation hazard assessment for additive manufacturing [15]. 

One of the more challenging and complex areas of AM are in the emerging field of gastronomy, or in other words, “3D Food printing” [4]. The ability to selectively deposit material within a 3D volume and, hence, gradate the composition offers the possibility of controlled production of complex structures for altering texture, taste, and morphology in food products. Manipulation of microstructures by regulating the mixing and selective deposition of materials can allow regulation of fracture, breakdown, or dissolution mechanics during product use, which gives the possibility of a range of functional and novel foods. 

In the past few years, several reviews highlighted different aspects of 3D Food printing [33]. For example, an overview of current research in printable food formulations [34,35], the review of 3D printing techniques applied to design food materials [36,37], the potential legal challenges and environmental implications of 3D Food printing [38], a review on existing food 3D printers alongside their advantages/disadvantages [39], and a review on 3D model design, model building, and model slicing in 3D Food printing [40]. However, the field of printable food materials has been growing, and, therefore, there is a need to collate and categorise published reports and to consolidate these developments. As such, we can have a better understanding of the accomplishments to date, and potential areas for future studies. In this paper, we will review the journal and conference proceeding reports on printable food materials. Section 2 discusses the additive manufacturing methods and materials in general. Section 3 explains 3D food printing and its advantages and discusses the reported materials for 3D food printing. The paper concludes with the future outlook and the potential research areas of AM in the food sector.

## 2. Additive Manufacturing: Material and Methods

Various 3D printing processes have been reported to date [41]. A summarised timeline of the history of AM can be found in Jakus (2019) [42]. The American Society of Testing and Materials (ASTM F2792-12a) have categorised the existing technologies under the following seven headings: (1) Vat photo-polymerization, (2) material extrusion, (3) directed energy deposition, (4) powder bed fusion, (5) binder jetting, (6) material jetting, and (7) sheet lamination [41,43]. These can be further categorised by the characteristics of the printed medium (liquid-, solid-, and powder-based materials) and, by the process, used to fuse matter on a molecular scale (thermal, UV-light, laser, or electron beam) [13]. These are described briefly in Table S2. To differentiate themselves, various manufacturers often use different acronyms for describing the same process, and, therefore, similar techniques might have different names [43]. These have all been previously compared and reviewed comprehensively [17,44,45,46], and many of these techniques have been individually reviewed in high detail including powder-based electron beam additive manufacturing (EBAM) technology [47], and the Fused Filament Fabrication (FFF) process, also termed, Fused Deposition Modelling (FDM) [48,49].

Materials have long been a barrier to the broader uptake of AM in the manufacturing industry [50]. There are several comprehensive reviews of 3D printing techniques in terms of the materials utilised among them [9,45,51,52,53,54,55]. The material selection depends on the process and the physical state of the material [13] as well as on the phase transitions or chemical reactions to bind the layers together [56,57]. The basic materials in 3D printing include a wide range of plastics such as commonly used acrylonitrile butadiene styrene (ABS), polylactic acid (PLA), and nylon. A handful of other materials, for example, cellulose plus its derivatives [58] and hydrogels [59], are also well established in AM and well accepted in some manufacturing applications. However, not many have reached the stage of full commercialisation.

Further progress in 3D printing will require the concurrent development of pioneering 3D printing methods in addition to novel feedstocks of material suitable for the devised processing methods [51].

## 3. 3D Food Printing

There are reports that focus on the effect of 3D-printed surface features on taste perception as a consequence of product form. For example, 3D-printed cups with angular surfaces increased perceived coffee and chocolate intensity ratings for bitterness and taste, but 3D-printed cups with rounded surface features cause a lower taste intensity and increase in sweetness perception [60]. Similar results have been reported on the effects of 3D-printed surface textures and visual design on ice-cream assessment [61]. For this kind of AM application in the food sector, all the materials and methods in Table S2 are potentially viable.

However, for the focus of this review, 3D food printing is the application of AM in food processing. It is one of the newest developments in food design and manufacturing with the potential for further studies and applications in the industrial sector. To date, five techniques (out of seven categories in AM, Table S2) have been used in 3D Food printing: material extrusion, powder bed fusion, vat polymerisation, binder jetting, and inkjet printing [34,57,62]. The same process principles, for AM in general, also apply to 3D Food printing. However, different degrees of pre-processing, such as fine-tuning of food recipes, and post-processing, such as cooking and oven drying, might be necessary for 3D food printing [4]. 

Some major challenges with 3D food printing include safety and labelling [38,63]. For example, some 3D printed samples, when stored in air, exhibited a high microbial concentration, which suggests extra consideration for hygienic equipment design is likely needed [11]. The average consumer does not favour 3D printed foods and typically holds a negative outlook associated with the fear of eating “alien foods” and a particular objection to foods that appear to have undergone a lot of processing [64,65].

However, many implications are mentioned to accompany 3D printed foods. The main potentials of AM technology in printing “edible materials” consist of:

- *Resolving food shortage by reducing food waste and increasing the usage of existing food materials and by simplification of production and streamlining the supply chain*. The technology prevents food wastage by utilising unpreserved fruits and vegetables and low-value by-products (ex. meat off-cuts, which traditionally goes to waste) to create pleasant, wholesome food products. The company Upprinting Food [66] uses food waste as the “ink” for 3D food printers. The necessary ingredients like bread, fruits, and vegetables from residual food flows are blended and combined to produce a puree, which will later be seasoned by herbs and spices. The puree is then 3D printed, and these prints are baked and dehydrated so that the resulting product is nicely crunchy and long durable. Moreover, once established, 3D food printing eliminates many of food manufacturing processes (ex. shaping, baking). Therefore, food producers can change their focus from “food production” to “food ingredients” production [38]. Additionally, 3D food printing can make food transport more feasible. Special food “cartridges” designed with 3D printing in mind will have far longer shelf lives as well as specially tailored nutritional profiles [3], which make them especially suitable for use in developing regions [4]. NASA uses dry powders for 3D printing that can have a shelf life of up to 30 years [3].
- *Treating malnutrition by personalising/customising food.* One solution for the malnutrition problem is to provide each person precisely the nutrition they need. 3D food printing permits the creation of new geometries and offers original production ideas in food manufacturing with superior control over composition, structure, texture, and taste [67]. 3D food printing could provide a solution to reconfigure a specialised supply chain intended to assist people with special dietary requirements [68]. By combining the creation of uniquely textured foods with superior nutritional value [69] or ingredient combination during printing or using a multi-printing process that uses several ingredients [11], 3D food printing can lead to new directions in a domestic cooking or catering services [68]. Personalising/customising food, both in terms of sensory and nutritional profiles for special consumer groups (young people, the elderly, pregnant women, athletes, etc.) [11], can fix malnutrition problems.
- *Lowering environmental impact*. 3D food printing is almost a zero-waste production because only the food that is printed will be consumed. Furthermore, due to its flexible nature, 3D food printing will promote the incorporation of low-carbon food ingredients (algae, insects, etc.) into pioneering edible objects [68]. 3D printing seafood and meat from cells (here is a review on 3D printing of meat [70]), or plant protein resources with similar taste and texture to the original leads to the development of foods at reduced environmental impact and improved quality [2].
- *Eliminating redundant businesses*. During and after the complete transition to the period when everybody can 3D print their food at their own home, some culinary professions, as well as food market-related jobs, will be eliminated [38].
- *Alleviating issues surrounding ‘food on the go’ to astronauts and military personnel [63] or ‘food as pharma’ in hospitals (for humans) or veterinary clinic (for pets).* The only necessary things to make the food will be food cartridges, food 3D printers, and energy [38].

### Materials for 3D Food Printing

Table 1 summarises chronologically the reported 3D food printing material and methods currently being employed. Although there is not a universal definition for food or even edibility, the main question in 3D food printing is the development of edible printable materials [71]. It is suggested that this review can be seen as a first step to identify and bring together the current disparate attempts to 3D-print "food.”

For 3D food printing to become practical, precise calibration of printing parameters should be carried out, dictated by the mechanical properties of the material. Furthermore, the study of the relationship between the rheological characteristics and their connection with printing parameters is key for improving the overall quality of 3D printed food [2]. There are still shortcomings in the knowledge of how to link material structure to process printing parameters to produce a desirable 3D printed product [4]. In previous years, two significant areas within 3D food printing have been investigated: (i) study of the relationship between 3D food printing and mechanical properties of food as well as rheological properties [72,73], and (ii) the formulation and development of 3D food printing products. However, most of the time, a clear border cannot be drawn between these two areas. 

In general, 3D printable materials must exhibit a controllable viscoelastic response, must form stable structures capable of withstanding compressive stresses from capillary forces, and must not shrink too much when undergoing drying, to avoid deformation and/or fissure formation [58]. These materials must be able to hold their shape once deposited. They need to be printable into defined shapes without slumping, spreading, or bridging. Shape fidelity assesses how well the printed structure conforms to the original design [73]. It is prevalent to further classify the materials into natively printable (i.e., confectionery, dairy, hydrogels) versus non-natively printable (i.e., plants, meat) [31,34]. However, this cannot be accurate for several reasons: (i) there is no consensus on how to assess or predict shape fidelity, let alone printability [74]. (ii) Printability is affected by multiple factors (temperature, components and additives) [75]. (iii) Various ingredients (varying in flavour and nutritional value) can be printed at once by using multiple cartridges. (iv) One material can be printable in one technique and non-printable in another technique.

As seen in Table 1, the most suitable materials for 3D food printing are carbohydrates, fats, proteins, fibre, and functional components [35]. Usually, there are several hydrogels that are popular in the field of 3D printing, including alginate, gelatin, chitosan, methylcellulose, agar, and carrageenan. Generally, hydrocolloids are used for fine-tuning material properties and to improve printability [3]. Apart from the materials reported in Table 1, there exist other 3D printed food products created by developers, companies, and designers looking to test the capabilities of the technology. Examples include an extruded pizza base topped with tomato sauce and cheese [76], rose-shaped pasta, rose-shaped chocolates, and sugar confections in geometric shapes [65]. 

As seen in Table 1, the extrusion method is the most widely adopted [32]. To be used in extrusion-based printing, a material should display shear thinning behaviour. This is an indicator that the material can be extruded from a nozzle [73]. One advantage of extrusion-based printing is the wide variety of food materials that can be extruded from a nozzle. These are then able to hold their shape and support the weight of the next layer [73]. Currently, extrusion-type 3D printing is used to prepare many different types of food such as dough [2], mashed potatoes [4], cheese [19], and meat [70] with a variety of complex and unique structures.

In inkjet printing, the drop deposition process consists of two independent stages of ejection and detachment. Hence, the material properties require calibration with the printhead design in mind. This is to avoid the presence of undesirable “satellite drops” in the breakup pattern [77,78]. Besides pneumatic stress, electrostatic attraction between the ink and the substrate can aid jetting. Inkjet printing has been widely approved for food decoration design by the EU and the US Food and Drug Administration (FDA). There exist commercial printing systems and inks for this application [65,78].

For binder jetting, the most widely available material is sugar. There are also reports on 3D-printed cereals created via selective laser sintering (SLS) [77].

In the following information, we have listed the most important milestones from Table 1, based on our views, in chronological order.

In 2009, a fundamental work in 3D food printing was carried out by Vesco et al. [79] to create a wide range of textures (i.e., mouthfeels). They believed the feedstock for 3D food printing must be developed in such a way that enables the 3D printing of an extensive range of foods without expanding unnecessarily the size of the required materials, which was set to be kept at a manageable level. They developed two-component basic hydrogel formulations of gelatin and xanthan and investigated their mouth-feel in sensory analysis. By varying the concentrations of the two gelling agents, it was possible to cover a matrix of textures ranging from weak nongranular materials simulating milk to firm nongranular (chocolate, mushroom) and solid granular materials (tomato). This method gave them many degrees of freedom in texture and flavour while comprising a minimal number of required materials.

Therefore, they established one way to generate texture variety in products with a bread-like structure that could be the fractionation of individual bread-structuring functions into modular formulation components (e.g., viscosity, bubble-stabilisation, structure), which was followed by recombination at varying ratios [80].

Based on these promising results of tailor-fitted food textures in an attempt to use 3D food printing for making “traditional” food, in 2010, Lipton et al. [81] 3D printed cereal paste or protein pastes (meat or scallop). They investigated the additives (transglutaminase or agar) effect on shape fidelity of the respective 3D printed structures before and after postprocessing by cooking or deep frying. Transglutaminase allowed the meat to be directly 3D printed for the first time by widening the library of material. This work demonstrated that 3D printed food could be prepared like traditional cuisine.

The implementation of alternative ingredients could help alleviate a potential global food shortage. 3D food printing has been employed to design appropriate insect products as a new source of proteins to overcome the disgust of consumers by consuming whole insects. An example of 3D printing technology applied to edible insects is represented by Soares and Forkes [82] in 2014, who printed the flour made out of edible dried insects in combination with fondant to produce icing for top cakes’ decoration. They focused on creating visually appealing products that would appeal to consumers. They used shapes inspired by insects’ natural form and movements, such as their wing patterns and eggs when formulating the design of these food products, attempting to make the shapes resemble jewelry rather than mimicking existing food products. Further work was carried out by Severini et al. [83] to obtain snacks from insect-enriched wheat flour dough as a new source of proteins. The printed snacks reproduced the overall structure of the designed object, but the addition of different concentrations of the insect powder modified the printability of the dough and the morphological and microstructural properties of the final product. However, the nutritional quality was higher than that of the un-supplemented wheat dough. After being baked, the size of the snacks was reported to be decreased due to water loss. Nevertheless, the enrichment of dough with insects reduced this effect. Moreover, baking conditions modified their microstructure, which induced non-enzymatic browning reactions and altered mechanical properties and protein digestibility [83].

In 2015, Inkjet technology found application on microencapsulation processes. The Netherlands Organisation for Applied Scientific Research (TNO)’s experience in inkjet printing suggested that the ability to generate monodisperse droplets might be of interest to food sectors. Their invention reports a printhead (500 nozzles with a capacity of 100 L/h) that produces highly monodisperse droplets converted into highly monodisperse powders after drying. Their printing technology allowed the use of a wide range of materials (waxes and fats, polymers, aqueous (or other) solutions, emulsions, and dispersions). Core-shell microcapsules and particles were prepared to encapsulate aqueous flavour and colourant solutions. Moreover, they fabricated calcium alginate gel particles made by printing alginate drops through a screen of calcium chloride solution [84,85].

In 2018, Vancauwenberghe et al. [86] designed a co-axial extrusion printhead to deposit a pectin-based ink and Ca^2+^ cross-linking solution in the inner and outer flows, respectively. This design facilitates an accurate control over the textural properties and gelation of the printing object as well as eliminates the pre-treatment or post-treatment step. In their earlier study [87], they had formulated the food-inks by changing the pectin, sugar syrup, or bovine serum albumin (BSA) concentration to obtain printed objects with the variable texture and structural properties. They had found that pectin and sugar concentrations were affecting the build quality of the printed objects (by changing the viscosity of the food-ink) and were improving the mechanical properties while BSA was stabilizing and promoting the porosity of the gel. In another attempt to eliminate the post-processing step, Hertafeld et al. [88] integrated infrared cooking into the 3D food printer to be able to extrude and cook simultaneously.

Again in 2018, by using a commercially available processed cheese as the printing material, Tohic et al. [89] assessed and compared the texture and mechanical properties of untreated, melted, and printed cheese samples. They found that the 3D printed cheese samples are different than melted and untreated samples in bulk mechanical properties. They concluded during the printing process that fat globules disrupt (during shearing) but partially coalesce (during solidification), which leads to the softer structure of 3D printed samples.

For 3D printed foods that do not require post-processing treatment (e.g., cooking, baking), such as mashed potato, chocolate, and cheese, the studies commonly assess how the rheological and mechanical properties [90,91] of food materials or the printing parameters (nozzle size, printing speed) [2,92] affect the printability. Few studies have taken into account the effect of post-printing treatment on the 3D printed samples. However, given the highly perishable nature of most food materials, the post-printing feasibility is necessary. In 2018, Lille et al. [93] used a food material set of cold swelling starch, milk powder, rye bran, oat, and faba bean protein concentrates and cellulose nanofiber for 3D printing. Moreover, after measuring the hardness and dry matter content, they compared the effects of oven drying (100 °C for 20–30 min) and freeze-drying (at −18 °C) on the shape fidelity of the 3D printed samples. Their results show that freeze-drying has almost no effect on the shape fidelity of the 3D printed samples. In oven drying, high water binding capacity reduces the drying rate and, therefore, better shape fidelity after drying.

The infill pattern refers to the structure that is printed inside the 3D construct. Structural properties of the 3D object by varying infill structure has been investigated mostly in polymer and bio-printing. In polymer printing, it has been reported that the honeycomb pattern is relatively tough with an increase in infill density (20%, 50%, and 100%) as compared to rectilinear and line patterns [94]. In bio-printing, a well-developed inner structure in 3D tissues construction such as a cross-link pattern is essential to maintain the mechanical properties of the constructs [95]. There are also few reports on designing an internal structure of 3D constructs to modify the textural properties of the printed foods. The textural and structural quality of mashed potatoes was investigated [96] by changing infill percentages (10%, 40%, 70%, and 100%) with different infill patterns (rectilinear, honeycomb, and Hilbert curve) and variation in shell perimeters (3, 5, and 7 shells). Although a direct relationship has been observed between the infill percentage and hardness, gumminess, and Young modulus of the printed samples, no similar results have been obtained for the infill pattern. Most recently, the 3D printing extrusion method has been used to make cylinders out of chocolate with various infill patterns and void percentages using powdered chocolate [97]. Three different infill patterns (star, honeycomb, and the Hilbert curve) with a variation of infill percentages of 5%, 30%, 60%, and 100% for each infill pattern have been investigated. Star and honeycomb patterns showed a high mechanical property due to the crisscross integrated design.

It has been reported that the printed object with 100% infill has a lower breaking resistance than that prepared by the casting method. This might be due to the formation of empty spaces during the deposition of layers. In 2019, Mantihal et al. presented three samples of chocolate printed in a honeycomb pattern with infill percentages of 25%, 50%, and 100% [98] to 30 semi-trained panelists. The panel preferred their 3D printed chocolate with 25% infill because of its relative softness. A comparison of a cast chocolate sample with a 100% infill printed chocolate sample showed an equal preference for both samples.

From the works collected, it is clear that, even though studies have been steadily carried out over the last ten years, there have been few sequential linked developments of complexity and understanding of the chosen formulations. From this, an important factor in future uptake and advancement would be to focus on further developing these materials for more bespoke products and more detailed understanding.

## 4. Future of AM in the Food Industry

When 3D printing matures as an industry, it will likely find applications in the area of food science and technology. These niche food applications will draw from the power of the platform as a tool for individualised food design or customised manufacturing. Moreover, patentability could be another motivation for the industry to adopt 3D food printing.

Although there is still a challenge of consumer acceptance, of not only 3D printed foods but the incorporation of 3D food printers into the home [99], global food companies are investing in research into 3D food printing as well as utilising it in production [100,101]. Several 3D food printers are available for domestic use [101]. One company, called Natural Machines, created the Foodini 3D printer, which can make 3D printed food from fresh ingredients [43]. Companies releasing 3D food printers for the home envision that they will one day be considered as a standard kitchen appliance [65]. Most of the studies focus on improving the appearance of the printed food rather than developing technology that will give access to large-scale production systems [102].

In the food sector, there is some skepticism about the use or value of 3D food printing technologies to people’s lives [103]. There still exist many questions about the safety and capabilities of 3D food printing and how to address them [99]. At present, there are still many problems with 3D food printing that needs to be solved: (i) scale of 3D printing as a whole is a slow set of technologies, which often requires hours to produce large constructs. To be used on an industrial scale, the printers would either need to be faster, or sufficiently cheap to allow for thousands of them to be run simultaneously. However, the small scale food manufacturing industry (bakeries and chocolatiers) will benefit from the added artistry and customization through 3D printing. (ii) Environmental impact: 3D food printers are capable of positively impacting the environment by decreasing food waste and food or food-related transports as well as by producing/replacing meat. However, there is a downside as well. 3D food printing, if omnipresent, will change the agricultural practice and lead to a dramatic impact on the entire ecosystem [38]. (iii) Cleaning machines afterwards: it is crucial to establish a proper cleaning protocol to ensure food products are safe to eat. One food does not affect the next one to maintain high quality over the lifetime of the 3D printers.

One step, before full transition to 3D food printing, could be adopting hybrid technologies, i.e., 3D printing methods adapted to current food manufacturing processes. How the technology can leap from the novelty sector will depend on the capability to identify a broader range of edible feedstocks, which is something that is being done in academia but will require input from both the layperson in their kitchen and from printer developers in the industry [104].

**Table 1:** Printable food materials.

Entry	Material	Printing Method	Printer	Year [Ref]	Comments-Major Finding
1	Mixed hydrocolloid systems (Xanthan gum and gelatin) and flavourings	Extrusion (cold)	Fab@Home printer	2009 [79]	They have shown that it is possible to create an extensive range of textures and tastes by mixing two hydrocolloids and flavour additives. However, such controlled food is typically reserved for medical and space applications.
2	Milk chocolate	Extrusion (hot melt)	Custom designed in the house extrusion system	2010 [105]	The key parameters for chocolate 3D printing have been identified (the nozzle aperture diameter, the optimum nozzle height from the build bed, the extrusion, and the axis movement speed). However, they have used a screw-driven extrusion system, which is less common these days in 3D printing.
3	Modified turkey, scallop, celery using transglutaminase, a modified traditional cookie	Extrusion (cold)	Fab@Home printer	2010 [81]	To integrate 3D food printing into conventional kitchens, the printed object needs to retain its shape during post-printing processes such as cooking and frying. There are two critical methods for solving the shape stability problem: additives and recipe control. Here, for the cookie, the concentration of ingredients have been changed systematically to identify a processable and printable formulation. However, the content of some components has been increased up to 100% when compared to the traditional recipe. On the other hand, by adding transglutaminase to the meat puree right before printing, cross-links the proteins present in the meat and gives rise to a self-supporting structure.
4	Mashed potato, chocolate, and cream cheese	Extrusion (cold and hot melt)	RapMan 3.1	2011 [106]	Testing and comparing three methods (moulding, extrusion, and binder jetting) using a range of foodstuffs and edible products. The authors have pointed out an advantage of 3D printing to moulding that has rarely been addressed. Weak food structures cannot successfully be taken out of the mould.
5	Combinations of caster, icing, and silk sugars as well as blends with maltodextrin	Binder jetting	CandyFab	2011 [106]	
6	Insect flour made more acceptable through combination with other foods including icing butter, chocolate, cream cheese, and spices	Extrusion		2014 [82]	Health and preference are two main reasons to adopt customized food [107]. Considering the high nutritional value of edible insects [108], the authors have 3D printed visually appealing food to enhance the “preference” of a diet that may have beneficial effects on human “health.”
7	Mint syrup, wax, Linseed oil, carrageenan shell	Inkjet	TNO’s encapsulation printer	2015 [84,85]	Final products are: mint syrup core capsule with a wax shell (200 µm) and linseed oil capsule with a carrageenan shell (280 µm) Because of the use of Inkjet technology, the produced droplets are highly monodispersed. With a capacity of 100 L/h, this method of droplet generation can compete for the microfluidic-based methods easily.
8	Edible ink (made up of water, ethanol, glycol, and/or glycerol as solvents and an edible colourant.)	Inkjet	Various	2016 [109]	Inkjet printing is predominantly used for 2D graphical decorating, surface filling, or cavity depositing. It may be more appropriate to classify this as a 2D printing technique rather than a truly free form method of creating 3D edible objects.
9	Wheat dough	Extrusion (cold)	3D Printer mod. Delta 2040 (Wasp project, Italy) equipped with the Clay extruder kit 2.00 (Wasproject, Italy)	2016 [2]	Post-printing: Cooking The effects of layer height and infill on the printing quality of cereal-based snacks with a cylinder-like shape, before and after cooking, has been studied. Many of the 3D printers, which are used in 3D food printing research studies, are exclusively optimized for synthetic materials (such as PLA). Therefore, they might be unable to keep a good equilibrium between different printing parameters when the original ink is replaced with another material with different rheological properties.
10	Pavlova (mainly egg white) with chocolate garnish	Extrusion (cold)	EnvisionTEC GmbH Bioplotter	2017 [110]	Post-printing: Baking The authors have briefly raised the effect of the printing bed surface on the quality of 3D printing. This subject has rarely been addressed or systematically studied in 3D food printing.
11	A formulation of low methoxylated pectin, CaCl_2_, bovine serum albumin (BSA), edible colourant, and sugar syrup	FDM (cold)	Custom designed in the house extrusion system	2017 [87]	Post-treatment to solidify the pectin into a gel with Ca^2+^ ions. Pectin and sugar concentrations affect the build quality of the printed objects by changing the viscosity of the food-ink and improving the mechanical properties. At the same time, BSA stabilises and increases the porosity of the gel.
12	Dark chocolate with the addition of magnesium stearate	Extrusion (hot melt)	PORIMY 3D chocolate printer, China	2017 [111]	Magnesium stearate plays the role of flow enhancer by minimising the slippage in the extruder [112] during deposition, and, thus, better ‘printability’. Moreover, it changes the snap properties of the final product.
13	Dark cooking chocolate	Extrusion (hot melt)	Custom designed syringe-based extrusion	2017 [113]	Chocolate is one of the most commonly used material in 3D food printing due to its capacity to be printed via various methods and popularity in the high-end food market. In hot melt extrusion printing of chocolate, the crucial factors are particle size, degree of crystallinity, melting behaviour, and material composition. The optimum temperature for forming the most stable crystalline chocolate melt is 32 °C.
14	A formulation of low methoxylated pectin, CaCl_2_·2H_2_O, and red food colourant E122	FDM (cold)	Custom designed in house extrusion system equipped with a coaxial extrusion printhead)	2018 [86]	Creating a co-axial extrusion printhead has been used to deposit a pectin-based ink, and CaCl_2_ cross-linking solution in the inner and outer flows, respectively. Therefore, the initial incubation or post-treatment step has been eliminated. This is allowing more accurate control of object gelation and texture.
15	Vegemite and Marmite	Extrusion (cold)	the BioBot printer	2018 [114]	This work, as well as Reference [82], are indicative of the application of 3D food printing as an educational tool during outreach activities. These works are stressing on the entertaining and indulgence aspect of 3D food printing to create fun designs suitable for celebratory events such as birthday parties. The flow consistency index and the flow behaviour exponent have been indicated as the critical parameters in determining whether a material is ideal for 3D printing or not. To date, the rheological property or properties and their quantitative limits that dictate 3D printability have not been univocally identified [73].
16	Dough with varying quantities of water, sucrose, butter, flour, and eggs	Extrusion (cold)	PORIMY Co. Ltd., China	2018 [92]	The authors have reported that the dough formulation affects its viscoelastic properties and, thus, its printability. However, the conclusion is not surprising. There is a strong link between the additives and the rheological properties of dough (and gluten) [115].
17	Mashed potato	Extrusion (cold)	Shiyin Co. Ltd., China	2018 [96]	These studies give an insight into the following: (i) The link between the rheological properties of mashed potato and its printability, (ii) The effect of additives (gums and starch, strawberry juice) on the rheological properties of the mashed potato and its printability, and (iii) Using a dual extrusion printer to print objects with different layered structures.
18	Mashed potato modified by the addition of potato starch	Extrusion (cold)	FSE2, Bolimai Co. Ltd., China	2018 [91]	
19	Mashed potato mixed with gums of xanthan, guar, k-carrageenan, and a k-carrageenan- xanthan gum blend	Extrusion (cold)	CSE 1, Bolimai Co. Ltd., China	2018 [116]	
20	Mashed potato/strawberry juice	Dual extrusion (cold)	Shiyin Co. Ltd., Hangzhou, China	2018 [117]	
21	Processed cheese	Extrusion (hot melt)	Custom designed syringe-based extrusion	2018 [89]	This is the first report on 3D printed cheese and investigating the effect of several variables on its main textural and melting properties. The authors have concluded that 3D printing substantially affected the properties of cheese, especially being less hard and easier melt, which is due to the different fat particle size distribution. They have not started with fresh milk as raw material and, therefore, consider a full phase transition from sol (milk) to gel (cheese). They have not analyzed the reproducibility of the 3D printing of their samples. They have also observed a colour change in the samples extruded at different speeds. The effect of 3D printing on the final colour of samples is debatable and needs to be studied among different consumer preferences.
22	Starch, milk powder, cellulose nanofiber, rye bran, oat protein concentrate, and broad bean protein concentrate and their mixtures	Extrusion (cold)	VTT’s micron-scale dispensing environment based	2018 [93]	Post-printing: oven drying or freeze-drying It was found that the cellulose nanofiber (CNF) improves the shape stability of the printed structures (maybe due to its shear-induced alignment properties) and decreases the hardness of the dried objects. However, as the authors have also reported, formulations with a high amount of CNF, clog the nozzle, which might be caused by some larger fibre particles remaining in the CNF after fibrillation, or the shear-induced flocculation of the material when forced through the small tip of the nozzle [58].
23	Blend of orange concentrate with added vitamin D, wheat starch, and hydrocolloid/s (gum arabic, guar gum, k-carrageenan gum, and xanthan gum)	Extrusion (cold)	SHINNOVE-D1, Shinnove Co. Ltd., China	2018 [67]	Starch is an excellent binder and widely used as a thickening agent in the food industry. The best printability and mechanical strength have been obtained at the presence of κ-carrageenan. It might be because of the binding of carrageenan to the double helix structure of amylose/amylose in starch [118].
24	A mix of hydrocolloids and three types of powdered vegetables: broccoli, spinach leaves, and carrots	Extrusion (cold)	A YL-CUBE 3D food printer (YOLILO Co., Ltd., Korea)	2018 [119]	Xanthan gum with its high hydration ability can stop the expansion of the vegetable powder particles so that the rheological value of the gel system before and after the powder addition does not change significantly, and the difference of printability between different vegetable powders is reduced.
25	Amorphous powdered cellulose and a binder based on xanthan gum	Binder jetting	Dimatix DMP-2831 inkjet printer (FujiFilm, USA)	2018 [120]	Because of very limited feedstock, in the food sector, binder jetting has been used more for entertaining and as a hobby. It is one of the few research reports on the application of binder jetting in the food sector where xanthan gum has been used as a glue to “clump” the amorphous cellulose. The aqueous ink and enough heating, recrystallize (in a semi-crystalline form) the amorphous cellulose in the printed piece but the surrounding powder remains unbound and still amorphous.
26	Pectin combined with a food formula (banana, white canned beans, dried non-fat milk, lemon juice, dried mushrooms (B. Edulis), ascorbic acid.	Extrusion (cold)	3D Printer mod. Delta 2040 (Wasp project, Italy) equipped with the Clay extruder kit 2.00 (Wasproject, Italy).	2018 [121]	This work is studying the implementation of 3D printing technology to provide innovative 3D snacks based on fruits by targeting a customized food formula for children from 3 to 10 years old. It is an example of the application of 3D food printing to overcome the malnutrition by getting people to snack five fruits/vegetables a day. Lemon juice and ascorbic acid, have been used to avoid the enzymatic browning of a printable food formula consisting mainly of banana.
27	Wheat flour dough with additive (calcium caseinate) containing probiotic	Extrusion (cold)	ByFlow, the Netherlands	2018 [122]	Post-printing: oven baking This report, as well as its similar report in Reference [123], are excellent examples of the application of 3D food printing in resolving malnutrition. The survival of a microorganism in baked food is low because of the high baking temperature. Usually, the rate of drying of the products is increased to shorten the baking time. Therefore, the product should have a high surface to volume ratio, which is very common in 3D printed samples.
28	Lemon juice gel with a range of potato starch concentrations	Extrusion (cold)	Custom designed in house extrusion system	2018 [124]	Proposing a mathematical formula for extrusion rate
29	Wheat dough enriched with ground yellow mealworms larvae (Tenebrio Molitor, an edible insect)	Extrusion (cold)	3D Printer mod. Delta 2040 (Wasp project, Italy) equipped with the Clay extruder kit 2.00 (Wasproject, Italy).	2018 [83]	Post-printing: oven baking See entry 6
30	Liquid chocolate	Inkjet	Custom designed in house electrostatic inkjet system	2018 [125]	The authors have used the electro-spraying technology (referred to as electrostatic inkjet method), which utilises electrostatic force to print gel-like materials, with a high-precision.
31	Combination of three different starches (potato, rice, and corn starch)	Extrusion (hot melt)	HE-3D printer SHINNOVE S2, China	2019 [126]	The extrusion process breaks the crystallinity and structure of the starch molecule. The disruption of the crystalline structure breaks the intermolecular hydrogen bonds during the gelatinization process. This is one of the parameters that leads to a formation of continuous starch paste matrix of entangled amylose molecules with suitable viscoelastic property and shear-thinning behavior [127].
32	Epoxidised vegetable oils	SLA	Custom designed in house apparatus	2019 [128]	This is where 3D food printing can help decrease the food waste. It is possible to transform the used cooking oil from McDonald’s into 3D printing resin [129].
33	The mixtures of soy protein isolate, gelatin, and sodium alginate	Extrusion (hot melt)	Custom designed syringe-based extrusion	2019 [7]	Soy protein isolate gel has a very high viscosity. Its rheological properties are not suitable for 3D printing. Similar to entry 19, hydrocolloids have been used for further tuning the viscoelastic properties.
34	A paste made out of brown rice and food additives (guar gum and xanthan gum)	Extrusion (cold)	Shiyin Co. Ltd., China	2019 [130]	See entry 19
35	A paste of beef gelatin, sucrose, egg white protein powder, and starch from corn	Extrusion (hot melt)	PORIMY3D Printing Technology Co., Ltd., China	2019 [131]	Studying tribological properties results in an improved understanding of rheological properties, therefore, in optimizing printing formulations.
36	Heat-induced egg yolk paste	Extrusion (cold)	SHINNOVE-E1, SHIYIN Technologies Co. Ltd., Hangzhou, China	2019 [132]	This is another method for using 3D printing in developing protein resources. Heating egg yolk leads to a three-dimensional protein network with a hard, cohesive, rubbery texture.
37	Cake icing, Hershey’s cocoa powder, Hershey’s chocolate syrup, and Nutella hazelnut chocolate spread	Extrusion (cold)	SHOTmini 200 Sx and IMAGE MASTER 350 PC Smart, Musashi Engineering Inc., Japan	2019 [133]	This approach bypassed a significant requirement of temperature control to perform 3D printing of chocolates by hot-melt extrusion. The presented technology offers an easy route to fabricate 3D structures of chocolate-based inks with liquid fillings using multiple dispensers.
38	Anthocyanin-potato starch gel Lemon juice-potato starch gel	Extrusion (cold)	SHINNOVE-D1, Shinnove Co. Ltd., Hangzhou, Zhejiang, China	2019 [134]	This is an example of moving from 3D food printing to 4D food printing. 4D printing refers to the response of a 3D printed object to stimuli from the environment, which results in physical or chemical changes in state over time. In this case, the color of the 3D printed samples is changing over time. It can be applied to prepare more visually appealing food products.
39	Sesame paste, chicken paste, and shrimp paste	Extrusion	Custom designed syringe-based extrusion	2019 [88]	Integrating an infrared lamp heating mechanism into the printer allows extruding and cooking food products simultaneously with high precision.
40	Potato starch	Extrusion (hot)	SHINNOVE-S2 printer Shiyin, China	2019 [135]	Similar to entries 18–20
41	Dark chocolate	Extrusion (hot)	Shinnove 3D printer (model no. Shinnove-D1, Shiyin Co. Ltd., Hangzhou, China)	2019 [98]	3D printed chocolates were fabricated by varying the infill structures (infill patterns and percentages) for textural and sensorial evaluations. A comparison of a cast chocolate sample with a 100% infill printed chocolate sample showed an equal preference for both samples, which are partly influenced by their perceived texture.
42	Egg yolk and egg white with blends of rice flour	Extrusion (cold)	Delta type 3D food printer CARK - Controlled Additive-manufacturing Robotic Kit	2020 [136]	Rice flour (1:1 and 1:2 w/w) plays the role of a filler agent and, therefore, has a significant effect on the improvement of stability and strength of printed materials with egg yolk and egg white.
43	A mixture of 50% native wheat starch, 40% maltodextrin, and 10% palm oil powder	SLS	EOS P380 machine	2020 [137]	Maltodextrin and palm oil play the role of the binder. It was obtaining a constitutive model that describes the large-strain material behaviour of 3D printed starch-based foods based on careful experimental research.
44	Two types of dark chocolates, Magnesium stearate, or plant sterol	Extrusion (hot melt)	PORIMY 1.0 (PORIMY, Kunshan, China)	2020 [97]	The star and honeycomb infill pattern produced the most stable and tough structure at 60% infill.

There exist three types of extrusion printing: screw-based extrusion, air pressure driven extrusion, and syringe-based extrusion [7,35,56,114,138,139]. In this table, screw-based extrusion in which the material is fed to the machine through a hopper, is called an extrusion system. FDM, Fused deposition modelling. SLA, stereolithography. SLS, selective laser sintering. FDM is the extrusion system where the feed is entering the nozzle via a tube, and a syringe pump drives it. Hot-melt extrusion is when the feed enters the extrusion in a temperature higher than the ambient temperature against cold extrusion where the feed is at an ambient temperature.

## Supplementary Materials

The following are available online at https://www.mdpi.com/2304-8158/9/4/497/s1. Table S1: Generic advantages and shortcomings of AM. Table S2: Additive manufacturing methods.
