# Supplementary material for: How to Formulate for Structure and Texture via Medium of Additive Manufacturing-A Review
Source: Foods. 2020 Apr 15;9(4):497. doi: 10.3390/foods9040497 (PMC7231001; doi:10.3390/foods9040497)
Supplement: Supplementary file 1 [file foods-09-00497-s001.zip › foods-760622-supplementary.docx]

Supplementary material

How to Formulate for Structure and Texture via Medium of Additive Manufacturing- A Review

Azarmidokht Gholamipour-Shirazi ^1,^*, Michael-Alex Kamlow ^1^, Ian T. Norton ^1^ and Tom Mills^1^

^1^ School of Chemical Engineering, University of Birmingham, Edgbaston, Birmingham, B15 2TT, UK

***** Correspondence: a.g.shirazi@bham.ac.uk;

Received: date; Accepted: date; Published: date

Table S1 Generic advantages and shortcomings of AM [1–3]

| Advantage | Economic effect |
| --- | --- |
| AM is capable of efficiently creating complex geometric product features resulting in higher performance levels. | Enables the production of highly functional and multifaceted products. |
| AM confers the capability to flexibly manufacture small product batches, down to a single unit in a cost-effective manner. | Small scale product customisation is economically attractive compared to traditional means of mass-production. Means of saving on cost include 1) Reduced demand for expensive tools, moulds or punches 2) Automated production 3) Usage of widely available supplies 4) Improved recyclability of waste material 5) Putting an end to unsold finished goods inventories 6) Enhanced working capital management due to payment being received before manufacture of goods 7) Distribution facilitating direct interaction between consumer/client and producer on a local scale |
| AM makes the sharing of design and outsourcing of manufacture far more feasible. | Risk of issues with regards to intellectual property, especially copyright infringement risk |
| AM yields improvements to the time taken and ease of delineating products. Digital files can be easily shared, enabling easy customisation of components and products for the user. | The tailoring of products to the individual users or function is a way of effectively producing highly customised products providing greater utility to end-users. The same equipment producing varied parts directly from digital files means no use of moulds and/or tools, greatly reducing switch over costs. |
|  |  |
| Shortcoming |  |
| The restricted palette of available materials and colours | The use of non-standard materials incurs additional costs, either due to inherent material properties or price |
| Slow process speed. | Increased knock-on costs due to time-dependent stages of AM |
| Poor dimensional accuracy and lower precision compared to some means of conventional manufacturing. | Possible requirement to use expensive and time-consuming post-processing requirements. |
| Coarse finish, limited product strength, heat resistance, moisture absorption and colour stability | As above, there is a possible requirement to use expensive and time-consuming post-processing requirements. |
| Difficulties with process repeatability and predictability. | Could lead to further costs due to building failure and quality. |
| Cost-effectiveness - Higher costs for large scale manufacture compared to injection moulding and other technologies | Unfavourable process economics at medium to large production runs compared to conventional manufacturing techniques |

*Table S2 Additive manufacturing methods*

|  |  | Ink type [4–7] | Process | Advantage [5,8–10] | Drawbacks [5,8–10] |
| --- | --- | --- | --- | --- | --- |
| Material Jetting | DOD | Polymers | A print head deposits droplets of liquid on to a surface. These droplets set spontaneously. The most common types of DOD print heads are thermal or piezoelectric actuation.[11,12] | Fast and efficient, complex structure production, high precision, easily adaptable with multiple nozzles to facilitate multi-material printing, rapid gelation enables swift layer-by-layer build-up. | High maintenance, high cost and short service life, require low viscosity ink (3–12 mPa.s), poor thermal conductivity, small volume droplets is a restriction on any potential scale-up, printing vertical 3D structures are not straightforward |
|  | NPJ | Polymers | A print head selectively deposits droplets of liquid on to a surface. They are then fused using a heat source.[12] |  |  |
|  | MJ | Polymers | A print head selectively deposits droplets of liquid on to a surface. They are then cured using ultraviolet (UV) light.[12] |  |  |
| Binder Jetting | BJ | Polymers, Gypsum, Sand | In this process, the utilisation of a binder causes layers of powders to be selectively bonded. Small droplets of the binder with radii less than 50 μm are deposited in succession on to the powder bed surface.[13] | Quick, simple and relatively inexpensive | Can only use powders, relatively weak mechanical characteristics |
| Powder Bed Fusion | SLS | Plastics, metals, alloys, metals with polymers and combinations of metals and ceramics. | Production consists of powder deposition followed by powder solidification. After this, the build platform lowers by the thickness of one layer. This three-step cycle repeats until the final layer has sintered. The sintering process utilises a laser source.[7] | No support structure needed, large range of material options, desirable mechanical properties, fine resolution, high quality | High power usage, size limitations, inefficient, limited quality, expensive, slow printing, highly porous |
|  | MJF | Polymers, ceramics, metal, | A fusing agent is jetted onto a heated powder bed alongside a second jet of detailing agent to improve part resolution, infra-red heaters pass over the bed sintering the powder at the fusing agent locations. |  |  |
|  | DMLS/SLM | Plastic, Stainless steel, Titanium, Aluminium, Cobalt, Chrome, Steel | Parts are produced through placement a thin layer of metal powder, followed by targeted lasing to achieve the pattern designed on the computer in that section. The lasing causes the particles to melt for less than a second allowing them bind to the existing structure below.[14] |  |  |
|  | EBM | Titanium, Cobalt, Chrome, Stainless steel, Aluminium, copper | The powder is melted by an electron laser beam powered by a voltage usually between 30 to 60 KV. The process occurs in a high vacuum chamber thus avoiding oxidation issues as it is intended for production of metal parts.[1,15] |  |  |

*BJ, binder jetting; CLIP, continuous liquid interface production; DLP, direct light processing; DMLS, direct metal laser sintering; DOD, drop-on-demand; EBAM, electron beam additive manufacturing; EBM, electron beam melting; FDM, Fused deposition modelling; LENS, laser engineering net shape; LOM, laminated object manufacturing; MJ, material jetting; MJF, multi jet fusion; NPJ, nanoparticle jetting; SLA, stereolithography; SLM, selective laser melting; SLS, selective laser sintering; SSE, semi-solid extrusion; UAM, ultrasonic additive manufacturing. The acronyms are taken from Awad et al (2018)* [12]

*Table S1 Additive manufacturing methods -continued*

| VAT Polymerisation | SLA | Liquid Photopolymers (acrylic or epoxy based) | Layer-by-layer manufacture by spatially controlled photopolymerisation of a liquid resin carried out by a scanning laser.[16] | High accuracy, high precision, high efficiency, good processing effect, low cost | Limited feedstocks, relatively costly, Lots of material used up as support structures, complex structure |
| --- | --- | --- | --- | --- | --- |
|  | DLP | Liquid Photopolymers (acrylic or epoxy based) | Layer-by-layer manufacture by spatially controlled photopolymerisation of a liquid resin carried out by a digital light projector.[16] |  |  |
|  | CLIP | Acrylates | A liquid resin undergoes continuous liquid interface production (CLIP) that uses a bottom-up building approach. It utilises a controlled oxygen inhibited dead-zone to prevent attachment of the part to a curing window that is oxygen-permeable.[17] |  |  |
| Material Extrusion | FDM | Thermoplastic filaments ( ABS, Nylon, PLA), Composites, Nano-fillers, concrete | A filament is heated until it is molten or semi-molten. The softened filament passes through a nozzle, with a solid filament acting as a piston. Then deposited onto the print surface. The deposited material then fuses with any adjacent material that has already been deposited.[18] | Inexpensive, uncomplicated, low maintenance, can produce relatively large constructs, room temperature processing, and high speed. | Gaps in the final product, intricate structures are difficult to produce, low accuracy, rough product surface, slow print speed, weak mechanical properties, limited materials |
|  | SSE | Gels and pastes | Extrusion of semi-solids focusing on applications for gels and pastes [11] |  |  |
| Direct Energy Deposition | LENS | Metals, Cobalt, Chrome, Graded/hybrid metals, Ceramics, Composites | A fabrication method where a high-powered laser is focused directly on the substrate creating a molten pool. Metal powder particles are then injected into this area to allow layer-by-layer buildup. The substrate is lowered beneath the laser beam, depositing a thin cross-section to produce the desired design. Further layers are repeatedly added to build a 3D part.[19] | Used for structural repair, multiple axes, flexible, high resolutions (pico- to microscale), can handle small printing volumes (pico-to-nanoliter) | Limited feedstocks, post-processing could be required, high cost, complex design |
|  | EBAM | Metals (powders/wires) | A high-energy electron beam is used as a moving heat source, to melt and fuse metal powder. Which then rapidly self-cools and produces parts in a layer-building manner. [20] |  |  |
| Sheet Lamination | LOM | Plastic, Metals, Graded/hybrid materials, Paper | Using sheets of the desired material a cross-section is cut into the sheet and is then attached to the cross-section of the part being made. A laser cuts the sheet of material that is spread across a movable substrate, and a laser cuts it along the contours of the part geometry designed on the computer. The layers are fused by a hot roller compressing the sheet and activating a heat-sensitive adhesive. [21] | High speed, low cost | Post-processing could be required, limited feedstocks |
|  | UAM | Plastic, Metals, Graded/hybrid materials | A combination of ultrasonic metal seam welding and CNC milling in the lamination process. UAM is the only AM technique that can construct metal structures at low temperature.[22] |  |  |

*BJ, binder jetting; CLIP, continuous liquid interface production; DLP, direct light processing; DMLS, direct metal laser sintering; DOD, drop-on-demand; EBAM, electron beam additive manufacturing; EBM, electron beam melting; FDM, Fused deposition modelling; LENS, laser engineering net shape; LOM, laminated object manufacturing; MJ, material jetting; MJF, material jet fusion; NPJ, nanoparticle jetting; SLA, stereolithography; SLM, selective laser melting; SLS, selective laser sintering; SSE, semi-solid extrusion; UAM, ultrasonic additive manufacturing. The acronyms are taken from* [12]

1. Baumers, M.; Dickens, P.; Tuck, C.; Hague, R. The cost of additive manufacturing: Machine productivity, economies of scale and technology-push. *Technol. Forecast. Soc. Change* **2016**, *102*, 193–201.

2. Berman, B. 3-D printing: The new industrial revolution. *Bus. Horiz.* **2012**, *55*, 155–162.

3. Ford, S.; Despeisse, M. Additive manufacturing and sustainability: an exploratory study of the advantages and challenges. *J. Clean. Prod.* **2016**, *137*, 1573–1587.

4. Garmulewicz, A.; Holweg, M.; Veldhuis, H.; Yang, A. Disruptive Technology as an Enabler of the Circular Economy: What Potential Does 3D Printing Hold? *Calif. Manage. Rev.* **2018**, *60*, 112–132.

5. Mitchell, A.; Lafont, U.; Hołyńska, M.; Semprimoschnig, C. Additive manufacturing — A review of 4D printing and future applications. *Addit. Manuf.* **2018**, *24*, 606–626.

6. Prakash, K.S.; Nancharaih, T.; Rao, V.V.S. Additive Manufacturing Techniques in Manufacturing -An Overview. *Mater. Today Proc.* **2018**, *5*, 3873–3882.

7. Ligon, S.C.; Liska, R.; Stampfl, J.; Gurr, M.; Mülhaupt, R. Polymers for 3D Printing and Customized Additive Manufacturing. *Chem. Rev.* **2017**, *117*, 10212–10290.

8. Lin, L.; Fang, Y.; Liao, Y.; Chen, G.; Gao, C.; Zhu, P. 3D Printing and Digital Processing Techniques in Dentistry: A Review of Literature. *Adv. Eng. Mater.* **2019**, *1801013*, 1–28.

9. Kyle, S.; Jessop, Z.M.; Al-Sabah, A.; Whitaker, I.S. ‘Printability’’ of Candidate Biomaterials for Extrusion Based 3D Printing: State-of-the-Art.’ *Adv. Healthc. Mater.* **2017**, *6*, 1700264.

10. Saptarshi, S.M.; Zhou, D.C. Basics of 3D Printing. In *3D Printing in Orthopaedic Surgery*; Elsevier, 2019; pp. 17–30 ISBN 9780323581189.

11. Boyd, B.J.; Jannin, V.; Goyanes, A.; Basit, A.W.; Vithani, K.; Gaisford, S. An Overview of 3D Printing Technologies for Soft Materials and Potential Opportunities for Lipid-based Drug Delivery Systems. *Pharm. Res.* **2018**, *36*.

12. Awad, A.; Trenfield, S.J.; Goyanes, A.; Gaisford, S.; Basit, A.W. Reshaping drug development using 3D printing. *Drug Discov. Today* **2018**, *23*, 1547–1555.

13. Pitayachaval, P.; Sanklong, N.; Thongrak, A. A Review of 3D Food Printing Technology. *MATEC Web Conf.* **2018**, *213*, 01012.

14. Zolfagharian, A.; Kouzani, A.Z.; Khoo, S.Y.; Moghadam, A.A.A.; Gibson, I.; Kaynak, A. Evolution of 3D printed soft actuators. *Sensors Actuators, A Phys.* 2016, *250*, 258–272.

15. José Horst, D.; Adriano Duvoisin, C.; De Almeida Vieira, R. *Additive Manufacturing at Industry 4.0: a Review*; 2018;

16. Waheed, S.; Cabot, J.M.; Macdonald, N.P.; Lewis, T.; Guijt, R.M.; Paull, B.; Breadmore, M.C. 3D printed microfluidic devices: enablers and barriers. *Lab Chip* **2016**, *16*, 1993–2013.

17. Stansbury, J.W.; Idacavage, M.J. 3D printing with polymers: Challenges among expanding options and opportunities. *Dent. Mater.* **2016**, *32*, 54–64.

18. Dudek, P. FDM 3D Printing Technology in Manufacturing Composite Elements. *Arch. Metall. Mater.* **2013**, *58*, 10–13.

19. Huang, S.H.; Liu, P.; Mokasdar, A.; Hou, L. Additive manufacturing and its societal impact: A literature review. *Int. J. Adv. Manuf. Technol.* **2013**, *67*, 1191–1203.

20. Gong, X.; Anderson, T.; Chou, K. Review on Powder-Based Electron Beam Additive Manufacturing Technology. *ASME/ISCIE 2012 Int. Symp. Flex. Autom.* **2012**, 507.

21. Guo, N.; Leu, M.C. Additive manufacturing: Technology, applications and research needs. *Front. Mech. Eng.* **2013**, *8*, 215–243.

22. Ngo, T.D.; Kashani, A.; Imbalzano, G.; Nguyen, K.T.Q.; Hui, D. Additive manufacturing (3D printing): A review of materials, methods, applications and challenges. *Compos. Part B Eng.* **2018**, *143*, 172–196.
